# Some Account of a Disease Lately Observed in Infants

**Published:** 1790

**Authors:** Thomas Denman

**Affiliations:** Licentiate in Midwifery of the Royal College of Physicians, London


X. *Some Account of a Disease lately observed in Infants. Communicated in a Letter to Dr.* Simmons *by* Thomas Denman, *M. D. Licentiate in Midwifery of the Royal College of Physicians, London.*

*To Dr.* SIMMONS.

DEAR SIR,

I REQUEST the favour of you to insert, in the London Medical Journal, the following notice of a disease (new perhaps in its manner, though not in its kind) which has proved fatal to several infants in the course of the present year.

The first symptom observed was that of the infants having a difficulty in breathing through the nose, a complaint which, in other seasons, is popularly called the *snuffles*, and is not suspected to be attended with any danger. This symptom appeared when the infants were about a fortnight old with so little deviation, that there was not a difference of more than three days in any one instance.

There soon followed a discharge from the nose, considerable in quantity, and varying in appearance, appearance, being ſometimes viſcid like mucus from an inflamed membrane, and ſometimes thin and ſanious, and mixed with a little blood.

The difficulty of breathing through the noſe was not conſtant, for there were intervals, even in the advanced ſtage of the diſeaſe, when the infants were almoſt wholly free from it, though at other times there were appearances which threatened inſtant ſuffocation; to prevent which an attendant watched the infants ſleeping or waking, to open their mouths when requiſite.

As the noſe was firſt affected, the complaint often ſeemed like a common cold, and when the infants breathed with tolerable eaſe, there was no ſign of danger; ſo that ſeveral erroneous prognoſtics were made by thoſe who were not acquainted with the nature of the diſeaſe.

Soon after the commencement there was a general fulneſs about the throat and neck externally, and a purple ſtreak at the verge of the eyelids, ſo ſingular, that after ſome experience, without farther inquiry, I knew, by the aſpect, the diſeaſe with which the infants were affected, and called it the *thruſh in the noſe*.

When theſe ſymptoms had continued for ſome days, according to the ſtrength of the infants fants and the degree of difeafe, they began to fwallow with difficulty, became pale and languid, and on looking into their throats, the doing of which properly requires fome addrefs, the tonfils were found tumefied and of a dark red colour, with afh-coloured fpecks upon them, and in fome there were extenfive ulcerations. The parts on which blifters had been applied in the beginning of the difeafe, and which had been apparently healed, often fphacelated towards the conclufion.

The infants gradually declined in their ftrength, and had a particular catch when they breathed, as if the *velum pendulum palati* was elongated: they were unable to fuck, though not univerfally; fwallowed with difficulty whatever was given in a fpoon, and died in convulfions, or with all the marks of great debility, though not on any particular day of the difeafe.

The firft cafe of this kind which I faw was in April. This infant was brought up by hand, but all the reft were fuckled. It recovered, though for many weeks it was fubject to fevere returns of the fnuffling in the nofe.

The fecond was in May. This infant was brought fick from the country, and died in convulfions convulſions on the third day after I ſaw it. The body was opened by Mr. Hunter and Mr. Home, but nothing was diſcovered except that the membrane which lines the noſe was of a dark red colour, and the blood veſſels were more turgid than ordinary.

The third was in June. This infant died; but not till more than three weeks from the time of its being taken ill.

The fourth was in July. This recovered.

The fifth was in Auguſt. This infant died with the throat very much ulcerated.

The ſixth was in Auguſt. This died.

The ſeventh was in September. This infant died on the fourteenth day of the diſeaſe; and it is remarkable that the wet nurſe and one or more of the ſervants had ſlight ſore throats at the time of its illneſs.

The eighth was in October. This alſo died.

In addition to the preceding account of the diſeaſe in queſtion, it may be obſerved that the ſeaſon has not been generally favourable to infants. Several have had inflammation and ulceration of the navel, and in one a gangrene came on which deſtroyed the infant in forty-eight hours. Three other infants have alſo died without any other tokens of diſeaſe except mere weakneſs, from which they could

 not not be raiſed. Many women have been brought to bed before their full time, but they have recovered well; and there has been no ſign of the puerperal epidemic which prevailed ſo frequently two and three years ago.

To give notice of a diſeaſe, without propoſing ſome method of treatment which has been found uſeful, does not ſeem of much importance; yet I am compelled to acknowledge that all which I was able to do, or which was recommended by others, gave me very little ſatisfaction: whether it was that the danger and nature of the diſeaſe were not at firſt ſuſpected or underſtood, or becauſe the degree of diſeaſe was beyond what the tender conſtitutions of infants were able to bear, or becauſe at ſo early an age infants ſubmit with ſuch diſadvantage to the application of medicine.

I take it for granted that no care could prevent the diſeaſe, as all who had it were the children of people of rank and fortune; nor was the common mode of treatment altered in any reſpect before they were taken ill.

The firſt child was ſuppoſed to have a common cold in an increaſed degree. Very little medicine was given, yet the child recovered. The death of the ſecond child was attributed to to convulfions. In the third the difeafe was referred to fome accidental caufe. The fourth child marked the difeafe, and it recovered, though very little medicine was given, and it might be lefs regarded on account of the dangerous ftate of the mother. The two fubfequent children dying, an alarm was given on the firft appearance of the difeafe, and active medicines were prefently ufed, but without fuccefs.

It is unneceffary to give a detail of the treatment of each cafe: I fhall, therefore, only obferve, that at firft the antimonial powder was given in fmall dofes, which commonly acted as an emetic; or faline mixtures, with teftaceous powders, magnefia, or fmall quantities of rhubarb. When the infants became weak, mixtures, with the pulv. contrayerv. comp., or confect. aromat., in decoction of bark, and other cordials, were given frequently, and in full dofes. Clyfters, compofed chiefly of bark, were adminiftered every four or fix hours. The wet nurfes alfo took bark, and were directed to live on a generous diet. Great care was taken to keep the paffage of the noftrils clear, and to prevent the humour which flowed from them from being fwallowed, by foliciting it outwardly, and by a favourable poſition of the child. The feet and legs were fomented, and emollient or gently ſtimulating cataplaſms afterward applied. Bliſters were alſo applied till their propenſity to become gangrenous was diſcovered. The noſe and throat were occaſionally fomented. In one caſe I gave the confect. aromatic. with decoction of bark the moment the diſeaſe appeared, but the child died.

Should any of your correſpondents have met with this diſeaſe, and have been ſo happy as to diſcover a ſucceſsful method of treatment, they would render an acceptable ſervice to ſociety by publiſhing it.

I am, dear Sir,

Your faithful humble Servant,

Thomas Denman.

*Old Burlington Street,*

Nov. 28, 1790.

